# A Relapsed AML Case Featuring MYC and MECOM Rearrangements

**DOI:** 10.3390/diagnostics15182410

**Published:** 2025-09-22

**Authors:** Kevin A. Murgas, Pons Materum, Luke Z. Li, Jacob Rocha, Michael Schuster, Tahmeena Ahmed, Carlos A. Tirado

**Affiliations:** 1 Medical Scientist Training Program, Stony Brook University Renaissance School of Medicine, Stony Brook, NY 11794, USA; kevin.murgas@stonybrookmedicine.edu; 2 Department of Pathology, Stony Brook University Hospital, Stony Brook, NY 11794, USA; 3 Department of Hematology and Oncology, Stony Brook University Hospital, Stony Brook, NY 11794, USA

**Keywords:** acute myeloid leukemia, relapse, cytogenetic analysis, chromosomal rearrangement, *KMT2A*, *U2AF1*, MYC, *MECOM*, *EVI1*, *ETV6*

## Abstract

**Background/Objectives:** Relapsed acute myeloid leukemia (AML) is often characterized by clonal evolution and acquired genomic abnormalities, which can inform prognosis and direct therapeutic decisions. The emergence of high-risk chromosomal rearrangements during relapse is of particular significance, yet the impact of rare and complex events remains poorly understood. This report details a case of relapsed AML that demonstrated rare *MYC* and *MECOM* rearrangements and additional features that were not observed at initial diagnosis, emphasizing the clinical relevance of serial cytogenetic assessments. **Case Description:** A 70-year-old man was initially diagnosed with AML, exhibiting monocytic differentiation, an 11q23 deletion involving *KMT2A* loss, and a *U2AF1* mutation. After achieving remission with azacitidine and venetoclax, the patient relapsed within ten months, necessitating reevaluation and modification of therapy. Repeat cytogenetic analysis at relapse revealed a distinct t(3;8)(q26.2;q24.3) exhibiting *MYC* and *MECOM* rearrangements, features that were absent at initial diagnosis. **Conclusions:** This case underscores the importance of serial cytogenetic and molecular profiling in relapsed AML. The emergence of new abnormalities upon relapse suggested underlying genomic instability and clonal evolution. *MYC* rearrangements are notably rare in AML, especially with concurrent *MECOM* rearrangements, highlighting a unique feature of this case. The identification of novel abnormalities at relapse may carry prognostic and therapeutic significance and may be used to refine risk stratification. Thus, ongoing cytogenetic monitoring is essential to adapt management approaches in evolving disease contexts.

## 1. Introduction

Acute myeloid leukemia (AML) is a heterogeneous group of leukemias that develop from dysregulated proliferation of myeloid cells, which can be driven by a broad variety of genetic mechanisms [1,2]. Genomic alterations are commonly observed in AML and may be readily detected by conventional cytogenetic techniques, including Giemsa-banded karyotyping and molecular cytogenetic techniques such as fluorescence in situ hybridization (FISH) and next-generation sequencing (NGS) [3]. The prognostic value of identifying these alterations motivates the routine use of cytogenetic assays to characterize each patient’s disease course and guide therapy [2,4]. Crucially, relapses of AML are often found to have acquired new cytogenetic abnormalities from the initial diagnosis, possibly representing unique clonal expansion that is selected for by cytotoxic therapy [5,6,7].

In this report, we describe a case of relapsed AML that developed a cytogenetic rearrangement involving *MYC* and *MECOM* (also known as *EVI1*) that was not present on initial diagnosis. Notably, *MYC* translocations are rare in AML, and concurrent *MECOM* rearrangements are even rarer, with only a few cases described in the literature; thus, this report may contribute insight into a distinct mechanism that drives AML relapse [8,9,10,11]. We summarize the clinical presentation, diagnostic workup, management, and surveillance, with an emphasis on the timeline of cytogenetic changes. We conclude with a discussion of proposed mechanisms of the genomic aberrations seen in this case, along with a review of the literature focusing on similar AML cases involving *MYC* and *MECOM* events.

## 2. Case Report

### 2.1. Case Presentation

**Figure 1:**
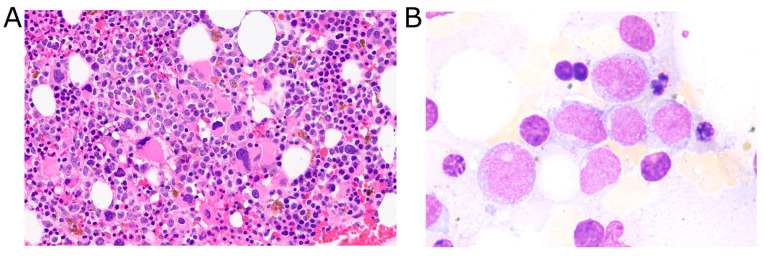
Bone marrow histology after AML relapse. (**A**): Representative image (40× magnification) of bone marrow core biopsy demonstrating increased blasts and hypolobated megakaryocytes. (**B**): Representative image (100× magnification) of bone marrow aspirate smear demonstrating blasts with abnormal morphology including large, moderate cytoplasm and vacuoles, along with features of erythroid dysplasia.

A 70-year-old man presented with six months of progressive exertional dyspnea. Initial labs revealed severe anemia with hemoglobin of 6.1 g/dL and moderate leukocytosis of 19.3 × 10^9^/L, prompting hospitalization and further workup. Peripheral blood smear showed increased monocytes, and flow cytometry also demonstrated a small population of circulating myeloblasts representing 0.3% of total events. Bone marrow biopsy demonstrated greater than 80% immature monocyte blasts on histology, diagnostic of AML with monocytic differentiation (Figure 1; Table 1). Additionally, prominent trilineage dysplasia was present. On flow cytometry, abnormal myeloid maturation with downregulated CD10 and CD16 was seen, and monocytes (65% of total) demonstrated aberrant downregulation of CD14 (34% of total). Cytogenetic analysis demonstrated a karyotype with chromosome 12p deletion in all cells and 11p deletion in a subset of cells, and FISH analysis detected *KMT2A* loss without gene rearrangement. NGS panel showed *U2AF1* c.101C>T (S34F) mutation with variant allele frequency of 50%. Notably, the *U2AF1* mutation indicated adverse risk based on the European Leukemia Network 2022 AML classification [2].

During the hospitalization, the patient’s anemia was managed with packed red blood cell transfusions. Given the patient’s significant debility, the patient was not felt to be a candidate for intensive induction and subsequently began treatment with a combination of azacitidine 75mg/m^2^ on days 1–7 and venetoclax 400mg daily in a 28-day cycle, with a ramp-up dose of venetoclax used. A repeat bone marrow biopsy after two cycles demonstrated complete morphologic remission, corroborated by flow cytometry finding no increased blasts or monocytes. Measurable residual disease was suggested by the presence of persistent *KMT2A* abnormality in a subset of cells. With his AML in remission, the patient continued this chemotherapy regimen for seven cycles. Given the risk of disease relapse, he was followed closely and was recommended for allogeneic bone marrow transplant but deferred during this time for personal reasons.

Ten months after diagnosis, the patient experienced recurrent fatigue; he was found again to be anemic, with hemoglobin of 6.0 g/dL. Repeat bone marrow biopsy revealed 14% CD34-positive blasts along with increased hypolobated megakaryocytes, signifying AML relapse. Molecular cytogenetic testing again demonstrated the previously seen loss of *KMT2A* and *U2AF1* mutation but also demonstrated *MYC* and *MECOM* rearrangements and loss of *ETV6*, findings that were not described at initial diagnosis. Collectively, these new findings indicated a complex karyotype with genomic instability and increased risk compared to the initial disease.

The patient was then initiated on high-intensity induction therapy with cladribine 5mg/m^2^ on days 1–5, cytarabine 20mg every 12 hours on days 1–5, and venetoclax 100mg on days 10–14, and was admitted for supportive therapy and transfusions. Post-induction bone marrow biopsy demonstrated residual disease, implying insufficient response to this regimen. The patient was then admitted for salvage reinduction with FLAG-IDA (fludarabine 30mg/m^2^ on days 2–5, cytarabine 2000mg/m^2^ on days 2–6, idarubicin 10mg/m^2^ on days 2–4, and filgrastim 300 mcg/m^2^ on days 1–5). Most recently, post-salvage bone marrow assessment demonstrated persistent AML. The patient remains transfusion-dependent and is undergoing enrollment for trial with cellular therapy.

### 2.2. Materials and Methods

#### 2.2.1. Conventional Cytogenetics

Chromosome analysis was performed using conventional cytogenetics protocols, including Giemsa (G-band) staining of metaphase cells. Results were annotated according to the International System for Human Cytogenomic Nomenclature 2024 standards.

#### 2.2.2. Molecular Cytogenetics

Fluorescence in situ hybridization (FISH) was performed with the XL MECOM (3q26) (MetaSystems, Medford, MA, USA), the LSI 5q EGR1/D5S23, D5S721 (5q31, 5p15.2), the LSI D7S486/CEP 7 (7q31/7p11.1-q11.1(D7Z1)), the LSI RUNX1/RUNX1T1(8q21.3, 21q22), the LSI MYC DC BAR (8q24), the LSI BCR/ABL1 DC DF (9q34, 22q11.2) (Abbott, Des Plaines, IL, USA), the XL KMT2A BA (11q23.3) the XL ETV6 BA (12q13.2) (MetaSystems, Medford, MA, USA), the LSI CBFB (16q22) DC BAR, and the LSI D20S108 (20q12) (Abbott, Des Plaines, IL, USA) probes. Probes were used following the manufacturer’s instructions for signal interpretation.

### 2.3. Cytogenetic Results

#### 2.3.1. Conventional Cytogenetics

At initial diagnosis, karyotype analysis of twenty cells revealed an abnormal male karyotype with segmental deletion at 12p13-12p11.2 in all cells and segmental deletion at 11q21-11q25 in nine of twenty cells, indicating evolution of a side clone containing two abnormalities. This karyotype was reported as 46,XY,del(12)(p13p11.2) [20]/46,XY,del(11)(q21q25),del(12)(p13p11.2)[9].

Upon relapse, a more complex karyotype emerged in a set of fifteen cells, including apparent translocations of chromosomes 3q26 and 8q24, in addition to the previously noted chromosome 11 and chromosome 12 deletions. This karyotype was reported as 46,XY,t(3;8)(q26;q24),del(11)(q21q25),add(12)(p13).

#### 2.3.2. Molecular Cytogenetics

At diagnosis, FISH was performed to examine two hundred interphase cells and metaphase cells using a comprehensive panel of commercial probes. FISH analysis revealed loss of *KMT2A* in 13.5% of cells, consistent with 11q23 deletion. Additional FISH markers were negative for common alterations involving *RUNX1/RUNX1T1* (t(8;21)(q22;q22)), *PML/RARA* (t(15;17)(q24;q21)), *CBFB* (16q22), *CSF1R/RPS14* (5q33), and *MDFIC* (7q31). Mutation testing for *FLT3*, *IDH1*, *IDH2*, *CEBPA*, *NPM1*, *PML::RARA*, and *cKIT* were also negative. The isolated finding of KMT2A loss was consistent with the del(11)(q21q25) reported by conventional cytogenetics. Further, a targeted NGS panel identified a *U2AF1* c.101C>T (S34F) mutation with variant allele frequency of 50%, which was denoted a Tier 1 variant of strong clinical significance. A variant of unknown significance with *KMT2A* c.7240T>A was also identified.

After two cycles of induction treatment, FISH analysis again demonstrated one copy of *KMT2A* in 28.5% of cells, while three copies of KMT2A were identified in 5.0% of cells, suggesting minimal residual disease that was not detected on histological and immunohistochemical examination or flow cytometry.

**Figure 2:**
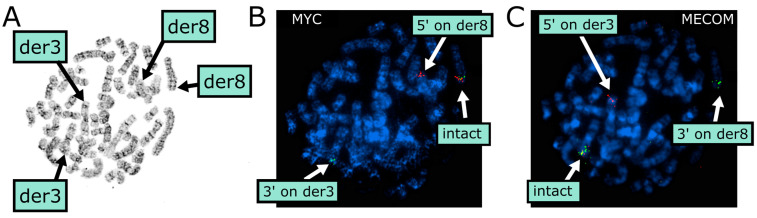
Rearrangement events involving *MYC* and *MECOM* diagnosed by FISH. (**A**): G-banded karyotype including identification of derivative chromosomes 3 and 8. (**B**): *MYC* break-apart FISH, showing one distal 3′ *MYC* probe (green) translocated to the derivative chromosome 3 with intact *MECOM* (overlapping red and green probes, shown in panel (**C**)). (**C**): *MECOM* break-apart FISH, showing one distal 3′ *MECOM* probe (green) translocated to the derivative chromosome 8, which had intact *MYC* (overlapping red and green probes, shown in panel (**B**)).

Upon relapse, additional cytogenetic changes were noted. FISH again demonstrated monosomy of *KMT2A* in 91.5% out of two hundred cells, suggesting expansion of the previously identified clones with *KMT2A* loss. Because conventional karyotyping suggested newly acquired chromosomal changes involving t(3;8)(q26;q24), break-apart FISH analysis on metaphase cells was essential to delineate the precise rearrangements of *MYC* and *MECOM* (Figure 2). Specifically, we observed a clone with *MECOM* rearrangement from one homolog of chromosome 3 onto one homolog of chromosome 8 with apparently intact *MYC*, evidenced by the 3′ (distal) signal of *MECOM* (green) on a derivative 8 that retained intact *MYC* and the 5′ (proximal) signal of *MECOM* (red) that was retained on one of the derivative 3; simultaneously, we observed *MYC* rearrangement from the other homolog of chromosome 8 to the other homolog of chromosome 3 with apparently intact *MECOM*, as evidenced by the 3′ (distal) signal of *MYC* (green) on the other derivative 3 that retained *MECOM* and 5’ (proximal) signal of *MYC* retained on the other derivative 8. These findings revealed that both copies of both chromosomes 3 and 8 were abnormal in this specific clone. Monosomy of *ETV6* was also present on FISH, consistent with 12p13 deletion. Additionally, NGS panel again identified a *U2AF1* c.101C>T (S34F) mutation with variant allele frequency 31.5%. Incorporating these FISH findings, the final karyotype of the relapse was reported as 46,XY,t(3;8)(q26.2;q24.3),del(11)(q23),add(12)(p13),der(3)(3′MECOM+,5′MECOM-),der(3)(3′MYC+),der(8)(5′MYC+,3′MYC-),der(8)(5′MECOM+).

## 3. Discussion

This case highlights the progressive nature of cytogenetic evolution in AML. At diagnosis, our patient had a deletion of 11q23 involving monosomic *KMT2A* loss without rearrangement, which is considered an intermediate-risk feature, and *U2AF1* mutation, which is considered an adverse-risk myelodysplasia-related gene mutation [2]. Despite initial rapid remission with cytotoxic therapy, the patient’s disease relapsed within a year. On relapse, the disease appeared to have undergone clonal evolution, retaining the *KMT2A* deletion and *U2AF1* mutation while acquiring several high-risk features in a subset of cells, including rarely observed concurrent rearrangements of *MYC* and *MECOM*. Critically, these features raised the possibility of genomic instability and clonal evolution driving leukemic progression and relapse. The relapse was treated with a shift in chemotherapy regimen, a strategy that was informed by emerging data suggesting that some patients with prior venetoclax exposure may still derive benefit from cytarabine-based reinduction regimens [4,12]. Given the acquisition of high-risk cytogenetics and insufficient response to the second-line therapy, the patient was subsequently transitioned to high-dose reinduction chemotherapy and was evaluated for further treatment options, including allogeneic hematopoietic stem cell transplantation as well as cellular-based therapy [4,13].

Mechanistically, this case implicates several genes with diverse mechanisms. The loss of one copy of *KMT2A*, a histone methyltransferase and tumor suppressor, likely contributed to the initial disease through activation of proliferation genes and disruption of the DNA damage response [14,15]. The proto-oncogene transcription factor *MYC* is often implicated as a central driver gene in AML, wherein *MYC* amplification is typically seen due to duplications, promoter mutations, or other gene-regulatory shifts, rather than chromosomal rearrangements [16,17,18]. Other studies have also implicated *PVT1*, a long non-coding RNA with a genomic locus that is directly downstream to *MYC*, as a key regulator of *MYC* activity involved in complex transcriptional feedback regulation, wherein disruption of local chromosome architecture may negate topological chromatin insulation, which normally prevents *MYC* activation by *PVT1* enhancer regions, thereby inducing *MYC* overexpression and driving proliferation [19,20]. Like *MYC*, *MECOM* is another transcription factor which can activate proliferation gene programs, and translocations of *MECOM* have recently been included as adverse risk factors in leukemia classification guidelines [2,21,22]. In our case, the distal 3q arm containing *MECOM* and distal 8q arm containing *MYC* and *PVT1* were rearranged to different homologs; a few prior cases of *MECOM::PVT1* rearrangement have been reported, though not in context of functional studies [10,23,24]. *ETV6* is another transcription factor involved in hematopoeisis, for which loss has been observed in AML [25]. Finally, *U2AF1* S34F mutation represents a specific splicing factor mutation that is known to be linked to poor outcomes [26,27].

### Review of the Literature

The phenomenon of clonal evolution is a well-established feature of relapsed and therapy-related AML, wherein selective pressure from prior cytotoxic therapy may lead to the emergence of new cytogenetic and molecular abnormalities. A review by Vosberg and Greif (2019) noted that a significant proportion of relapsed AML cases acquire new genetic abnormalities that are not present at initial diagnosis, thereby signifying underlying genomic instability and selection of resistant subclones [5]. Clonal evolution studies further support the view that such rearrangements may emerge during treatment, contributing to disease progression and therapy resistance [6,7]. Our case provides a striking example of this process, with the emergence of several high-risk genomic features at relapse.

Rearrangements involving *MYC* and *MECOM* represent rare but clinically meaningful events in AML, particularly in relapsed or therapy-related cases. *MECOM*, a complex gene locus encoding the *EVI1* transcription factor, is a well-established adverse prognostic marker associated with chemoresistance and poor survival [28,29,30]. *MYC* abnormalities are more commonly associated with lymphoid malignancies yet remain rare in AML [16,17]. When *MYC* rearrangements do occur in myeloid neoplasms, they frequently correlate with aggressive disease [31]. Although both *MYC* and *MECOM* rearrangements are individually recognized in hematologic malignancies, their co-occurrence in AML is exceedingly rare. The first reported t(3;8)(q26.2;q24) translocation in myelodysplastic syndrome (MDS) was identified in 1987 [32]. A review of the recent literature identified a small number of case reports and case series in which simultaneous rearrangements of the *MYC* and *MECOM* loci were documented (Table 2). These cases tended to emerge in the context of disease progression or therapy-related AML and were frequently associated with adverse-risk cytogenetic features.

Liu and Tirado (2018) reported a case of polycythemia vera progressing to AML and exhibiting t(3;8)(q26.2;q24) [8]. This finding was established by chromosome analysis and confirmed by break-apart FISH. The authors further queried the Mitelman Database of Chromosome Aberrations and Gene Fusions in Cancer to identify 36 additional cases of AML and 4 cases of MDS which harbored a t(3;8) translocation and examined common co-occurring abnormalities, the most common of which was monosomy 7. Furthermore, the authors highlighted an incomplete understanding of the molecular mechanism of this translocation in driving AML, noting that some cases did not exhibit *PVT1* involvement, as others had hypothesized [8,33].

Tang et al. (2019) reported twenty cases of t(3;8)(q26.2;q24) resulting in *MYC* and *MECOM* rearrangement in patients from a single institution, including six previously reported cases [9]. These cases, diagnosed over a 20-year period, encompassed therapy-related AML and MDS, as well as blast phases of chronic myeloid leukemia and myeloproliferative neoplasms. One-third of cases showed megakaryocyte hypoplasia, as in this case. Karyotypes were analyzed, and *MYC* and *MECOM* involvement were confirmed with break-apart FISH probes. Additional sequencing panel analysis identified a few cases with mutations in *ABL1*, *ASXL1*, *BRINP3*, *CBFB*, *GATA2*, *NRAS*, *RUNX1*, *SRSF2*, and *TET2*; notably, *FLT3* and *KRAS* mutations were not detected in any of these cases. The authors further characterized survival outcomes, noting a median overall survival of 6 months and inferior survival compared to a control cohort of 44 cases without t(3;8), indicating an aggressive disease subtype with poor outcomes [9].

Smith et al. (2020) reported two additional cases of MDS with confirmed *MYC* and *MECOM* rearrangements [10]. Both cases utilized break-apart FISH probes to diagnose *MYC* and *MECOM* translocations. The authors note that their *MYC* break-apart probe was insufficient to distinguish *PVT1* involvement; however, they reference a key study that demonstrated *MYC* and *PVT1* overexpression in cases with t(3;8) rearrangements to support the hypothesis of both genes being involved [23]. They additionally reviewed 33 previously reported cases in a 10-year retrospective search of the Human Genetics Laboratory database, encompassing AML, MDS, and blast-phase chronic myeloid leukemia. They again identified monosomy 7 in 45% of these cases, while noting a single occurrence of monosomy 5, which is more common in inv(3) and t(3;3) cases [34]. Mechanistically, the authors proposed that the translocation activated *MECOM* while disrupting *MYC* regulatory control by *PVT1*, contributing to disease progression [10].

In addition to FISH and sequencing techniques, recent advances in optical genome mapping (OGM) have enhanced the detection of subtle genetic rearrangements [11,35]. McGinnis et al. (2024) demonstrated this technology on 90 cases from a 10-month period encompassing AML and lymphoblastic leukemias [11]. The authors identified several rearrangements that standard cytogenetics, including FISH, failed to detect, even describing a *MECOM::MYC* rearrangement, although further specifics on the case or cases were not provided. This study emphasized the sensitivity of OGM for high-resolution genomic profiling to capture low-frequency or subtle-to-detect events, highlighting promising benefits from the evolving techniques of cytogenetic surveillance if practically implemented [36].

Lastly, emerging evidence suggests that AML with monocytic differentiation, as in this case, may respond poorly to venetoclax and azacitidine; a single-institution cohort of 86 AML patients distinguished poorer treatment response and survival in cases with greater than 10% monoblasts [37]. Cytogenetic information, including *U2AF1* mutation status, has been studied in AML to stratify and predict the response to venetoclax [38]. Notably, novel therapies are emerging in the field of AML, such as menin inhibitors for *KMT2A* rearrangements (not deletions as in this case) [39]. Formulated combination treatments such as liposomal CPX-351 (cytarabine and daunorubicin) show promise for AML cases with high-risk features [40]. Hence, the prognostic information provided by serial cytogenetic analysis may better guide management decisions, as well as support trials investigating alternative therapies in AML [41].

Collectively, the current literature provides insight into *MYC* and *MECOM* rearrangements as rare yet critical late-acquired events in AML, reflecting clonal evolution and conferring adverse prognosis. Detection of these genetic abnormalities is enabled by advanced cytogenetic techniques like FISH and OGM. Moreover, this line of research raises critical questions regarding the functional consequences of *MYC* and *MECOM* rearrangement, particularly whether these rearrangements represent cooperating leukemogenic events. Our case contributes to this sparse but growing literature by documenting concurrent *MYC* and *MECOM* rearrangements acquired at relapse and reinforces the diagnostic value of cytogenetic evaluation in identifying and monitoring complex cytogenetic events.

## 4. Conclusions

This case underscores the importance of serial cytogenetic and molecular characterization, particularly at relapse, where the emergence of new abnormalities can significantly alter prognosis and guide critical treatment decisions. In relapsed AML, especially with adverse-risk cytogenetics, prompt intervention with modified therapy based on prior treatment history is imperative. AML cases exhibiting complex karyotypes with genomic instability pose a distinct challenge and merit research attention to unravel the effects of co-occurring rearrangements and mutations. Thus, improved cytogenetics techniques and characterization will continue to enhance our clinical understanding of genomic alterations driving AML, ultimately working towards the goal of improving patient outcomes.

## Figures and Tables

**Table 1:** Timeline of key clinical events and pathological findings. Bone marrow histology, flow cytometry, and cytogenetics findings are summarized at key timepoints when bone marrow biopsies were performed. Percentage of cells with abnormalities is reported. Abbreviations: VAF variant allele frequency.

Time	Bone Marrow Histology	Flow Cytometry	Cytogenetics
0 months: initial diagnosis	>80–90% immature monocytes, trilineage dysplasia	Abnormal myeloid maturation, abnormal monocytes (65%)	del(12p) (100%), del(11q) (45%), *KMT2A* loss (13.5%), *U2AF1* mutation (VAF 50%)
3 months: post two cycles chemotherapy	No evidence of residual disease	No abnormal myeloid or blast populations	*KMT2A* loss (28.5%), *KMT2A* gain (5.0%)
10 months: relapse	14% blasts, decreased erythropoiesis, dysplastic megakaryocytes	Mild increase in myeloblasts (8%)	t(3;8), del(11), del(12), *MYC* (68%) and *MECOM* (18%) rearrangements, *KMT2A* loss (91.5%), *ETV6* loss, *U2AF1* mutation (VAF 31.5%)
15 months: post salvage reinduction	Minimal residual disease	Residual AML blasts 2–3%	t(3;8), del(11), *MYC* (16.5%) and *MECOM* (30%) rearrangements, *KMT2A* loss (93.5%)

**Table 2:** Summary of published cases exhibiting concurrent MYC and MECOM rearrangements. Summarized are the source publication, number of patients, abnormalities identified, age, sex, treatment(s), and outcome(s). Abbreviations: AWD—alive with disease; NS—not specified.

Source	N Patients	Abnormalities Identified	Age	Sex	Treatment(s)	Outcome(s)
Liu and Tirado 2018 [8]	1	t(3;8)(q26.2;q23)	68	Male	Ruxolitinib	Death
Tang et al. 2019 [9]	20	t(3;8)(q26.2;q24), various gene mutations	Range: 21–79 Median: 61	12 male, 8 female	Various	Death 90%, AWD 10%
Smith et al. 2020 [10]	2	Both: t(3;8)(q26.2;q24.1) Case 1: der(5), *IGH*/*CCND1* fusion Case 2: del(7), del(13q14), loss of *ASXL1*, *CSF3R*, *ETV6*, and *U2AF1*	60; 68	Both male	NS; Rituximab	Death; AWD
McGinnis et al. 2024 [11]	NS (at least 1)	*MECOM::MYC* rearrangement	NS	NS	NS	NS
Present case	1	t(3;8)(q26.2;q24.3), del(11)(q23), del(12)(p13), *ETV6* loss, *U2AF1* c.101C>T (S34F) mutation	70	Male	Venetoclax + azacytidine	In remission

## Data Availability

No new data were created or analyzed in this study. Data sharing is not applicable to this article.

## Abbreviations

AML—acute myeloid leukemia; FISH—fluorescent in situ hybridization; t(3;8)—translocation of chromosomes 3 and 8.
